# Seizures in brain tumor hospitalizations in the United States

**DOI:** 10.3389/fneur.2025.1680216

**Published:** 2026-02-12

**Authors:** Alka Mithal, Maanek Sehgal, Herbert Newton, Derek Ems, Vince Florio, Gurkirpal Singh

**Affiliations:** 1 Institute of Clinical Outcomes Research and Education (ICORE), Woodside, CA, United States; 2 Neuro-Oncology Program and Brain Tumor Institute, University Hospitals Cleveland Medical Center, Seidman Cancer Center and UH Neurological Institute, Cleveland, OH, United States; 3 UCB, Smyrna, GA, United States

**Keywords:** brain tumor, epilepsy, database analysis, National Inpatient Sample, seizures

## Abstract

**Introduction:**

A feasibility analysis was conducted using a national US hospitalization database to examine the prevalence and outcomes of concomitant seizures in brain tumor hospitalizations.

**Methods:**

All hospitalizations in adults aged ≥18 years with a primary/secondary diagnosis of brain tumor were studied from the National Inpatient Sample database from 2016–2020. Hospitalizations were classified into groups (with and without a recorded diagnosis of seizures [including epilepsy]).

**Results:**

Overall, 1,503,885 brain tumor hospitalizations were assessed. The prevalence of brain tumors varied throughout the study period, from 114–124 per 100,000 population. A total of 305,040 (20.3%) hospitalizations had concomitant seizures. Those aged ≥65 years had higher prevalence of hospitalizations with brain tumors. The Black population generally had the highest proportion of seizures in brain tumor hospitalizations across all study years, apart from 2017 when it was highest in the Native American population. For each study year, length of hospital stay was significantly longer and mean charges were significantly higher in brain tumor hospitalizations with seizures versus those without seizures. Case fatality was generally lower in brain tumor hospitalizations with seizures versus without seizures, and was significantly lower in 2016, 2017, and 2020.

**Discussion:**

Data from this analysis highlight differences in demographics and outcomes between patients with and without seizures and could be used to inform future analyses in this area.

## 1 Introduction

Brain tumors can develop as a primary tumor or as a metastasis of a solid malignancy from another location. The latter is the most common type of brain tumor, most frequently resulting as a complication of lung or breast cancer or melanoma (1). A reported 10–40% of solid tumors develop brain metastases, producing an estimated 70,000 to 400,000 cases of metastatic, or secondary, brain tumors per year (2). In 2019, the prevalence of primary brain or CNS tumors (malignant and non-malignant) was estimated at 1.3 million people in the US (3). In 2006, nearly 73,500 hospitalizations related to brain cancers (excluding secondary malignancies) were reported in the United States (4), while another study reported over 728,000 brain and nervous system cancer-related hospitalizations from 2008 to 2019 (5).

Brain tumors are associated with high morbidity and complications, including seizures (6). Patients with brain tumors frequently experience epileptic seizures, which are often the first clinical sign that a brain tumor is present (7). Seizure incidence is highly variable among patients with brain tumors, with estimates of concomitant seizures ranging from 20–80%, according to type and location of tumor (8). Seizures are most common in patients with glioneuronal tumors (70–80%, especially with lesions in the frontotemporal or insular regions), glioma (60–75% in low grade glioma located in superficial cortical or insular regions), meningioma (20–50%), and metastatic tumor (20–35%) (8). Studies suggest that new-onset seizure activity may be an early clinical indicator of a brain tumor and a good prognostic factor for survival (9, 10).

The mechanisms underlying brain tumor-related seizures are multifactorial and not yet fully understood (reviewed in (11–14)). Peritumoral regions become increasingly epileptogenic due to mechanical changes resulting from tumor growth, including localized tissue compression, ischemia, and metabolic changes such as acidosis and tissue damage (11). The pro-inflammatory state is characterized by glial cell and cytokine recruitment, which contributes to dysregulated blood brain barrier function and increased neuronal excitability (12, 13). Tumor-related alterations to inflammatory processes, gene expression, and signaling pathways increase seizure susceptibility (11, 14). Imbalances in neurotransmitters, with the overexpression of excitatory glutamate and inhibition of GABAergic input, and disruption of transport function, further contribute to epileptogenic susceptibility (11, 14). Once seizures manifest, treatment with antiseizure medication is recommended (15). However, management of both brain tumor and seizures can be challenging, with potential for drug interactions, adverse effects, impacts on quality of life, and risk of status epilepticus (16–20).

Scant data are available on the prevalence and outcomes of brain tumors and concomitant seizures in adults in the United States. In this feasibility analysis, the US National Inpatient Sample (NIS) database was used to assess the prevalence and outcomes of brain tumor hospitalizations with and without concomitant seizures. Data were stratified by sex, age, race/ethnicity, and insurer. Outcomes included mean length of hospital stay, case fatality, and mean charges. The analysis also allowed assessment of the NIS database for robustness of data of interest for more in-depth additional studies.

## 2 Methods

This study follows the guidelines for good pharmacoepidemiology practices (GPP) laid out in 2005 Federal Drug Agency GPP and the 2015 International Society of Pharmacoepidemiology (ISPE) Guidelines for GPP.

### 2.1 Data source

This database analysis used the publicly-available National Inpatient Sample (NIS) database, which is an approximately 20% stratified sample of US community hospital discharges (21). This large longitudinal database includes data from hospitals nationwide, ranging from 46 states plus the District of Columbia (DC) in 2016 to 48 states plus the DC in 2020. Thie NIS covers 98% of the US population and captures approximately 7 million hospitalizations per year (21). Discharge data included diagnosis codes (*International Classification of Diseases, Tenth Revision, Clinical Modification* [*ICD-10-CM*]), procedure codes (*ICD-10-PCS*), patient demographics, disease severity, payer, total charges, length of stay, and hospital characteristics. The database, which is maintained as part of the Healthcare Cost and Utilization Project, was designed to generate estimates of inpatient costs, utilization, and outcomes at the national level, with particular utility for analyzing special/uncommon populations, conditions, or treatments.

### 2.2 Identification of hospitalizations with brain tumor with and without seizures/epilepsy

For this analysis, all hospitalizations in patients aged ≥18 years with a primary or secondary diagnosis of brain tumor between January 1, 2016 and December 31, 2020 were identified using *ICD-10-CM* codes for brain tumor (primary and metastatic; Supplementary Table S1). Hospitalizations with brain tumors were classified into two groups according to whether there was a recorded primary or secondary diagnosis of seizures (including epilepsy), defined as the presence of ≥1 relevant *ICD-10-CM* code (Supplementary Table S2). All data were de-identified.

### 2.3 Outcomes

The prevalence of seizures among hospitalizations for brain tumors was assessed. The prevalence of brain tumor hospitalizations with concomitant seizures was calculated as a percentage of the total population hospitalized with brain tumors. Demographic variables were evaluated and described in groups with and without seizures for sex, age, race/ethnicity, and insurer. Other outcome variables included the mean duration of hospital stay (reported as total number of inpatient days divided by total discharges), mean hospital charges (reported as USD for each calendar year), and case fatality (reported for hospitalizations resulting in death as a proportion of total hospitalizations).

### 2.4 Statistical analyses

For hospitalizations with brain tumors with and without seizures, demographics and national estimates, standard error of the mean, and 95% confidence intervals were calculated using Statistical Analysis Software (SAS) program (version 9.4). Differences in outcomes for hospitalizations with brain tumor with and without seizures were compared using Welch T-tests.

## 3 Results

During the study period (2016–2020), there were approximately 300,000 hospitalizations per year with a primary or secondary diagnosis of brain tumors (1,503,885 hospitalizations overall).

### 3.1 Prevalence rates

Annual prevalence of hospitalizations with brain tumors in the US varied between 114 and 124 per 100,000 US population from 2016 to 2020 (Table 1). The ≥65 years age group had a higher prevalence of hospitalizations with brain tumors than younger age groups. Within the ≥65 age group, males had a higher prevalence than females. However, prevalence was higher in females in the younger age groups (18–44 and 45–64 years). Of hospitalizations with brain tumors, 305,040 (20.3%) had a concomitant primary or secondary diagnosis of seizures or epilepsy (Table 1).

**Table 1:** Prevalence of hospitalizations for brain tumor in the US population, and percentage of seizures or epilepsy in brain tumor hospitalizations.

	2016	2017	2018	2019	2020
Hospitalizations, *n* per 100,000 US population^a^					
Overall	116	119	121	124	114
Male	107	111	113	116	107
18–44 years	25	25	25	26	24
45–64 years	129	129	132	135	125
≥65 years	287	305	302	307	281
Female	124	126	128	132	120
18–44 years	35	36	36	36	34
45–64 years	152	152	152	155	142
≥65 years	270	273	277	283	254
Hospitalizations with diagnoses of interest					
Brain tumor, *n*	289,735	299,155	305,825	316,490	292,680
Seizures or epilepsy in those with brain tumor, *n* (%)	58,275 (20.1)	59,420 (19.9)	63,120 (20.6)	64,700 (20.4)	59,525 (20.3)

^a^Some hospitalization records have missing variables (e.g., sex, race).

Across all study years, patients hospitalized for brain tumors with seizures were younger on average than those without seizures (by approximately 3 years; Table 2). The 18–44-years age group had the highest percentage of concomitant seizures in both males and females. Within this age group, a higher proportion of males had seizures compared with females (32.7–35.8% for males, 21.3–22.8% for females; Table 2; See Supplementary Table S3 for 95% confidence limits). The ≥65 years age group had the lowest proportion of concomitant seizures (17.6–18.7% males, 16.2–17.7% females). A higher proportion of the Black population had concomitant seizures (22.9–23.9%) versus other racial/ethnic groups across all study years apart from 2017, when prevalence was higher in the Native American population (23.4% vs. 22.9%). A similar proportion of the White and Hispanic populations had concomitant seizures (~ 20%), while those from Asia/Pacific islands had the lowest proportion (15.4–16.5%) over the study period.

**Table 2:** Demographics in the US population hospitalized with brain tumor with and without seizures or epilepsy 2016–2020.

Variable	2016 (*N =* 289,735)		2017 (*N =* 299,155)		2018 (*N =* 305,825)		2019 (*N =* 316,490)		2020 (*N =* 292,680)	
	Seizures/epilepsy		Seizures/epilepsy		Seizures/epilepsy		Seizures/epilepsy		Seizures/epilepsy	
	With	Without	With	Without	With	Without	With	Without	With	Without
Hospitalizations, *n* (%)	58,275 (20.1)	231,460 (79.9)	59,420 (19.9)	239,735 (80.1)	63,120 (20.6)	242,705 (79.4)	64,700 (20.4)	251,790 (79.6)	59,525 (20.3)	233,155 (79.7)
Age, mean, years	59.8	62.9	60.3	63.4	60.5	63.6	60.4	63.9	60.8	63.9
Sex, *n* (% of subpopulation per calendar year)										
Male	28,375 (21.8)	101,640 (78.2)	29,575 (21.7)	106,420 (78.3)	30,980 (22.3)	108,155 (77.7)	32,830 (22.8)	111,310 (77.2)	29,970 (22.3)	104,225 (77.7)
18–44 years	4,820 (32.8)	9,895 (67.2)	4,750 (32.7)	9,775 (67.3)	4,935 (33.6)	9,750 (66.4)	5,470 (35.8)	9,795 (64.2)	4,695 (33.4)	9,380 (66.6)
45–64 years	12,580 (23.8)	40,195 (76.2)	12,410 (23.5)	40,470 (76.5)	13,300 (24.6)	40,780 (75.4)	13,610 (24.8)	41,370 (75.2)	12,270 (24.4)	38,100 (75.6)
≥65 years	10,975 (17.6)	51,550 (82.4)	12,415 (18.1)	56,175 (89.6)	12,745 (18.1)	57,625 (81.9)	13,750 (18.6)	60,145 (81.4)	13,005 (18.6)	56,745 (81.4)
Female	29,825 (18.7)	129,425 (81.3)	29,840 (18.3)	133,310 (81.7)	32,140 (19.3)	134,540 (80.7)	31,870 (18.5)	140,455 (81.5)	29,555 (18.7)	128,905 (81.3)
18–44 years	4,535 (22.7)	15,410 (77.3)	4,390 (21.3)	16,265 (78.7)	4,800 (22.8)	16,290 (77.2)	4,590 (21.6)	16,645 (78.4)	4,255 (21.3)	15,695 (78.7)
45–64 years	12,665 (19.4)	52,690 (80.6)	12,920 (19.8)	52,490 (80.2)	13,110 (20.2)	51,920 (79.8)	13,545 (20.5)	52,680 (79.5)	12,000 (20.0)	48,105 (80.0)
≥65 years	12,625 (17.1)	61,325 (82.9)	12,530 (16.3)	64,555 (83.7)	14,230 (17.7)	66,330 (82.3)	13,735 (16.2)	71,130 (83.8)	13,300 (17.0)	65,105 (83.0)
Race/ethnicity, *n* (% of subpopulation per calendar year)										
White	39,260 (19.7)	159,990 (80.3)	40,055 (19.4)	166,275 (80.6)	43,330 (20.5)	168,040 (79.5)	44,610 (20.2)	176,730 (79.8)	40,260 (19.8)	162,890 (80.2)
Black	8,275 (23.0)	27,765 (77.0)	8,560 (22.9)	28,850 (77.1)	9,520 (23.9)	30,335 (76.1)	9,630 (23.7)	31,070 (76.3)	9,140 (23.7)	29,370 (76.3)
Hispanic	4,650 (20.6)	17,915 (79.4)	4,630 (19.5)	19,170 (80.5)	5,195 (19.9)	20,915 (80.1)	5,135 (19.7)	20,930 (80.3)	4,960 (20.5)	19,250 (79.5)
Asian/Pacific Islander	1,505 (16.5)	7,590 (83.5)	1,575 (16.0)	8,250 (84.0)	1,625 (16.2)	8,400 (83.8)	1,695 (15.4)	9,305 (84.6)	1,690 (16.0)	8,870 (84.0)
Native American	215 (18.0)	980 (82.0)	280 (23.4)	915 (76.6)	240 (19.0)	1,020 (81.0)	240 (19.8)	970 (80.2)	280 (22.9)	945 (77.1)
Other	2,115 (23.1)	7,055 (76.9)	2,275 (22.2)	7,970 (77.8)	1,900 (19.2)	8,005 (80.8)	2,035 (22.0)	7,225 (78.0)	1,770 (21.0)	6,665 (79.0)
Insurance payer, *n*										
Medicare	26,740	115,350	28,200	123,745	30,215	125,910	30,645	132,215	28,575	121,005
Medicaid	8,395	27,335	8,100	28,450	8,465	29,180	9,070	28,875	8,240	27,875
Private insurance	19,920	76,090	19,820	74,755	20,930	74,540	21,345	76,715	19,240	71,170
Self-pay	1,325	5,195	1,375	5,415	1,620	5,665	1,735	6,335	1,690	5,640
No charge	155	495	120	410	95	415	115	435	90	490
Other	1,690	6,665	1,710	6,470	1,730	6,650	1,705	6,865	1,615	6,660

Medicare was the most frequent payer, followed by private insurer and Medicaid. Combined, Medicare and Medicaid paid for 60.3–61.8% of the brain tumor hospitalizations with seizures and 61.6–64.0% of the brain tumor hospitalizations without seizures.

### 3.2 Other outcome variables

Length of hospital stay, mean charges, and case fatality across groups and years are reported in Table 3. Length of hospital stay was significantly longer (*p* < 0.0001) and mean charges significantly higher (*p* < 0.05) in brain tumor hospitalizations with seizures compared with those without seizures across all study years. Case fatality was significantly lower in brain tumor hospitalizations with versus without seizures in 2016, 2017, and 2020 (all *p* < 0.05); no statistically significant differences were observed in 2018 and 2019.

**Table 3:** Charges, length of stay, and case fatality in the US population hospitalized with brain tumor with and without seizures or epilepsy 2016–2020.

Variable	2016		2017		2018		2019		2020	
	Seizures/epilepsy		Seizures/epilepsy		Seizures/epilepsy		Seizures/epilepsy		Seizures/epilepsy	
	With (*n* = 58,275)	Without (*n* = 231,460)	With (*n* = 59,420)	Without (*n* = 239,735)	With *n* = 63,120	Without (*n* = 242,705)	With (*n* = 64,700)	Without (*n* = 251,790)	With (*n* = 59,525)	Without (*n* = 233,155)
Charges, mean (SEM), US $	78,311 (1,937) *	72,214 (1,431)	81,396 (2,015) *	75,822 (1,468)	86,368 (1,793) *	80,367 (1,595)	92,074 (2,124) *	85,927 (1,660)	100,156 (2,336) *	93,599 (1,849)
95% LCL – UCL	74,512 – 82,109	69,409 – 75,019	77,446 – 85,345	72,944 – 78,700	82,854 – 89,883	77,241 – 83,494	87,909 – 96,239	82,672 – 89,182	95,577 – 104,735	89,974 – 97,224
Length of stay, mean (SEM), days	6.6 (0.1) ^‡^	6.0 (0.0)	6.6 (0.1) ^‡^	6.0 (0.0)	6.8 (0.1) ^‡^	6.0 (0.0)	6.7 (0.1) ^‡^	6.2 (0.0)	6.9 (0.1) ^‡^	6.3 (0.0)
95% LCL – UCL	6.5–6.8	6.0–6.1	6.4–6.8	5.9–6.1	6.6–7.0	6.0–6.1	6.5–6.8	6.1–6.2	6.7–7.1	6.2–6.3
Case fatality, mean (SEM), %	5.3 (0.2) ^†^	6.1 (0.1)	5.7 (0.3) *	6.3 (0.2)	5.6 (0.3)	6.1 (0.2)	5.6 (0.2)	6.0 (0.1)	5.6 (0.3) *	6.1 (0.1)
95% LCL – UCL	4.9–5.8	5.8–6.3	5.2–6.3	6.0–6.7	5.1–6.2	5.8–6.4	5.1–6.0	5.7–6.2	5.1–6.1	5.9–6.4

*p < 0.05; ^†^*p* < 0.01; ^‡^*p* < 0.0001. LCL, lower confidence limit; UCL, upper confidence limit.

## 4 Discussion

In this feasibility study, ~1.5 million brain tumor hospitalizations were assessed from the NIS over the study period (2016–2020) to gain insight on the prevalence and outcomes of hospitalizations with brain tumors and concomitant seizures. Brain tumor hospitalizations varied between 114 and 124 per 100,000 US population between 2016 and 2020. One-fifth of brain tumor hospitalizations had concomitant seizures. This concomitant seizure rate is somewhat lower than that reported in adults with metastatic brain cancer in a tertiary care center (approximately 30% of patients had chart-documented seizures) (22) and in patients with primary tumors admitted to an intensive care unit due to seizures (27%) (23). However, the seizure rate is higher than the 4% reported in a 2006 database study of US primary brain cancer hospitalizations with epilepsy/convulsion (4). Various study settings (single-center vs. nationwide database), design, and patient populations contribute to these differences. In this study, the approximately 20% prevalence of concomitant seizures with brain tumor hospitalizations captured seizures in both primary and metastatic brain tumors from a large nationwide inpatient database. Seizure activity in patients with brain tumors is highly variable and dependent on many factors, such as tumor histology, grade, and molecular phenotype (15). Although lower-grade tumors have higher incidence of seizures (15), hospitalizations tend to be weighted toward higher-grade malignant tumors. Of note in this analysis, a decrease in total brain tumor hospitalizations was observed between 2019 and 2020. Prior to 2019, the number of hospitalizations increased year on year from 2016 to 2019. This finding may be due to the impacts related to the COVID-19 pandemic, such as access restrictions to health care, insurer eligibility, changes to hospital policy, or disruptions in data reporting.

Age-, sex-, and race-related differences in brain tumor incidences are well documented (24–26), but there are scant data regarding potential differences for patients with brain tumors and concomitant seizures. In the current study, men aged 18–44 years had higher rates of concomitant seizures than women or other age groups. Seizures in brain tumor hospitalizations occurred for a higher proportion of the Black population versus other groups across all study years apart from 2017, when it was higher for the Native American population. A previous study using the Surveillance, Epidemiology, and End Results (SEER)-Medicare registry similarly reported increased risk of seizures for Black versus White patients with brain metastases (hazard ratio: 1.45 [95% confidence interval: 1.22–1.73]) (27). Such findings may suggest these patients presented with more severe disease or were subject to disparities in access to health care (27).

In hospitalizations with (versus without) concomitant seizures, longer length of stay and higher mean charges may partly reflect disease severity and efforts to manage seizures in a sicker population. Routine electroencephalography monitoring (with routine or continuous bedside monitoring to screen for subclinical seizures or epileptiform activities, or elective admission to an epilepsy monitoring unit to characterize clinical events or guide surgical decision-making), more time spent in a medical or neurological intensive care unit, and increased use of neurosurgical evaluations or procedures may contribute to longer stays and higher charges. In a retrospective study of patients with glioblastoma, seizures were the most common reason for acute care admission (28). The finding of lower mortality in the group with concomitant seizures has been observed previously in patients with brain tumors, specifically those with high-grade glioma (29). This is thought to be due to earlier diagnosis and treatment of the brain tumor (8), which could involve earlier use of computed tomography or magnetic resonance imaging scans, earlier surgical intervention (e.g., debulking, shunt placement), and earlier treatment of post-operative wounds and infections. Lower mortality could also reflect treatment of complications arising from other therapy options (e.g., radiotherapy, chemotherapy). These reasons may have contributed to the lower mortality observed in the population with brain tumor with (versus without) seizures in the current study.

Study limitations include this analysis providing association rather than causation. Data anonymization meant that a patient could be included more than once if they had subsequent hospital admissions. No information on seizure prophylactic use, seizure profiles, or tumor grade were available from hospital records. Tumor type, location, and malignancy status were not analyzed in this feasibility study. Furthermore, patients may have developed seizures or epilepsy after discharge from hospital, which would not be captured in this dataset. Database studies are subject to coding issues, such as errors, omissions, and incompleteness. As such, diagnostic codes may over (or under) count conditions, and the relevance of *ICD* codes to hospitalization may be over (or under) represented or confounded by clinical variables not captured in the database. For example, epilepsy as a chronic condition may not be recorded among the *ICD* codes in hospitalization records, thus underestimating its prevalence among the hospitalized population.

This study provides an update of the prevalence of brain tumor hospitalizations in the US, and of brain tumor hospitalizations with concomitant seizures. These findings demonstrated that the NIS, which is the largest publicly available, nationwide inpatient database, can provide vigorous data of interest. Observed age, sex, and racial differences may suggest possible disparities in health care. Data on outcomes suggest hospitalizations with brain tumors with (vs. without) concomitant seizures have lower mortality and incur longer hospital stays and increased costs, suggesting earlier treatment. Future analyses could assess the prevalence of epileptic seizures and their influencing factors in different types of brain tumors. An epidemiological overview could provide background information, such as tumor type and malignancy, treatment interventions and settings (community hospitals, academic hospitals, epilepsy units), further detail on reasons for admission (e.g., seizures alone, neurological deterioration, complications of chemotherapy), or antiseizure medications prescribed at discharge.

## Data Availability

Publicly available datasets were analyzed in this study. This data can be found at: https://hcup-us.ahrq.gov/db/nation/nis/nisdbdocumentation.jsp.
