# Supplementary material for: Seizures in brain tumor hospitalizations in the United States
Source: Front Neurol. 2026 Feb 12;16:1680216. doi: 10.3389/fneur.2025.1680216 (PMC12935659; doi:10.3389/fneur.2025.1680216)
Supplement: Supplementary file 1 [file Data_Sheet_1.pdf]

Supplementary Information

Seizures in brain tumor hospitalizations in the United States

Alka Mithal[1] | Maanek Sehgal[1] | Herbert Newton[2] | Derek Ems[3] | Vince Florio[3] | Gurkirpal Singh[1]

[1]Institute of Clinical Outcomes Research and Education (ICORE), Woodside, CA, USA

[2]Neuro-Oncology Program and Brain Tumor Institute, University Hospitals Cleveland Medical Center, Seidman Cancer Center and UH Neurological Institute, Cleveland, OH, USA

[3]UCB, Smyrna, GA, USA

Correspondence

Gurkirpal Singh, MD, Institute of Clinical Outcomes Research and Education (ICORE),
175 Eleanor Drive, Woodside, CA 94062, USA.

Telephone: +16505300020

Email: gsingh@stanford.edu

TABLE S1 *ICD-10-CM* codes used for diagnosis of brain tumor.

C700	C718	C7931	D352	D445
C709	C719	C7932	D354	
C710	C7232	D320	D420	
C711	C7240	D329	D429	
C712	C7241	D330	D430	
C713	C7242	D331	D431	
C714	C7250	D332	D432	
C715	C7259	D333	D433	
C716	C751	D337	D439	
C717	C753	D339	D443	

ICD-10-CM, International Classification of Diseases, Tenth Revision, Clinical Modification.

TABLE S2 *ICD-10-CM* codes used for diagnosis of seizures or epilepsy.

G40001	G40211	G4042	G40814	G40911	R5600
G40009	G40219	G40501	G40821	G40919	R5601
G40011	G40301	G40509	G40822	G40A01	R561
G40019	G40309	G40801	G40823	G40A09	R569
G40101	G40311	G40802	G40824	G40A11	
G40109	G40319	G40803	G40833	G40A19	
G40111	G40401	G40804	G40834	G40B01	
G40119	G40409	G40811	G4089	G40B09	
G40201	G40411	G40812	G40901	G40B11	
G40209	G40419	G40813	G40909	G40B19	

ICD-10-CM, International Classification of Diseases, Tenth Revision, Clinical Modification.

TABLE S3 Demographics in the US population hospitalized with brain tumor with and without seizures or epilepsy 2016–2020, including 95% upper and lower confidence limits.

	2016 (*N* = 289,735)		2017 (*N* = 299,155)		2018 (*N* = 305,825)		2019 (*N* = 316,490)		2020 (*N* = 292,680)	
	With seizures / epilepsy	Without seizures / epilepsy	With seizures / epilepsy	Without seizures / epilepsy	With seizures / epilepsy	Without seizures / epilepsy	With seizures / epilepsy	Without seizures / epilepsy	With seizures / epilepsy	Without seizures / epilepsy
Hospitalizations, *n* (%)	58,275 (20.1)	231,460 (79.9)	59,420 (19.9)	239,735 (80.1)	63,120 (20.6)	242,705 (79.4)	64,700 (20.4)	251,790 (79.6)	59,525 (20.3)	233,155 (79.7)
95% LCL – UCL	55,141 – 61,409	221,489 – 241,431	56,195 – 62,645	229,007 – 250,463	59,820 – 66,420	232,077 – 253,333	61,261 – 68,139	240,622 – 262,958	56,432 – 62,618	222,965 – 243,345
Age, mean, years	59.8	62.9	60.3	63.4	60.5	63.6	60.4	63.9	60.8	63.9
95% LCL – UCL	59.4 – 60.2	62.6 – 63.2	59.9 – 60.7	63.1 – 63.7	60.1 – 60.9	63.3 – 63.9	60.0 – 60.7	63.6 – 64.2	60.4 – 61.2	63.6 – 64.1
Sex, *n* (% of subpopulation per calendar year)										
Male	28,375 (21.8)	101,640 (78.2)	29,575 (21.7)	106,420 (78.3)	30,980 (22.3)	108,155 (77.7)	32,830 (22.8)	111,310 (77.2)	29,970 (22.3)	104,225 (77.7)
95% LCL – UCL	26,676 – 30,074	96,984 – 106,296	27,818 – 31,332	101,401 – 111,439	29,133 – 32,827	103,075 – 113,235	30,942 – 34,718	106,142 – 116,478	28,187 – 31,753	99,401 – 109,049
18–44 years	4,820 (32.8)	9,895 (67.2)	4,750 (32.7)	9,775 (67.3)	4935 (33.6)	9,750 (66.4)	5,470 (35.8)	9,795 (64.2)	4,695 (33.4)	9,380 (66.6)
95% LCL – UCL	4,304 – 5336	8,992 – 10,798	4,225 – 5275	8,874 – 10,676	4,412 – 5458	8,781 – 10,719	4,976 – 5964	8,900 – 10,690	4,183 – 5207	8,520 – 10,240
45–64 years	12,580 (23.8)	40,195 (76.2)	12,410 (23.5)	40,470 (76.5)	13,300 (24.6)	40,780 (75.4)	13,610 (24.8)	41,370 (75.2)	12,270 (24.4)	38,100 (75.6)
95% LCL – UCL	11,700 – 13,460	38,036 – 42,354	11,569 – 13,251	38,221 – 42,719	12,405 – 14,195	38,443 – 43,117	12,659 – 14,561	39,066 – 43,674	11,450 – 13,090	36,013 – 40,187
≥65 years	10,975 (17.6)	51,550 (82.4)	12,415 (18.1)	56,175 (89.6)	12,745 (18.1)	57,625 (81.9)	13,750 (18.6)	60,145 (81.4)	13,005 (18.6)	56,745 (81.4)
95% LCL – UCL	10,319 – 11,631	49,464 – 53,636	11,674 – 13,156	53,822 – 58,528	11,956 – 13,534	55,297 – 59,953	12,953 – 14,547	57,659 – 62,631	12,219 – 13,791	54,372 – 59,118
Female	29,825 (18.7)	129,425 (81.3)	29,840 (18.3)	133,310 (81.7)	32,140 (19.3)	134,540 (80.7)	31,870 (18.5)	140,455 (81.5)	29,555 (18.7)	128,905 (81.3)
95% LCL – UCL	28,187 – 31,463	123,829 – 135,021	28,187 – 31,493	127,348 – 139,272	30,500 – 33,780	128,729 – 140,351	30,132 – 33,608	134,174 – 146,736	28,037– 31,073	123,278 – 134,532
18–44 years	4,535 (22.7)	15,410 (77.3)	4,390 (21.3)	16,265 (78.7)	4,800 (22.8)	16,290 (77.2)	4,590 (21.6)	16,645 (78.4)	4,255 (21.3)	15,695 (78.7)
95% LCL – UCL	4,088 – 4982	14,262 – 16,558	3,941 – 4839	15,001 – 17,529	4,358 – 5242	15,087 – 17,493	4,122 – 5058	15,294 – 17,996	3,838 – 4672	14,559 – 16,831
45–64 years	12,665 (19.4)	52,690 (80.6)	12,920 (19.8)	52,490 (80.2)	13,110 (20.2)	51,920 (79.8)	13,545 (20.5)	52,680 (79.5)	12,000 (20.0)	48,105 (80.0)
95% LCL – UCL	11,857 – 13,473	49,884 – 55,496	12,097 – 13,743	49,724 – 55,256	12,291 – 13,929	49,187 – 54,653	12,690 – 14,400	49,912 – 55,448	11,230 – 12,770	45,537 – 50,673
≥65 years	12,625 (17.1)	61,325 (82.9)	12,530 (16.3)	64,555 (83.7)	14,230 (17.7)	66,330 (82.3)	13,735 (16.2)	71,130 (83.8)	13,300 (17.0)	65,105 (83.0)

95% LCL – UCL	11,904 – 13,346	59,068 – 63,582	11,789 – 13,271	61,997 – 67,113	13,467 – 14,993	63,835 – 68,825	12,947 – 14,523	68,381 – 73,879	12,574 – 14,026	62,596 – 67,614
Race/ethnicity, *n* (% of subpopulation per calendar year)										
White	39,260 (19.7)	159,990 (80.3)	40,055 (19.4)	166,275 (80.6)	43,330 (20.5)	168,040 (79.5)	44,610 (20.2)	176,730 (79.8)	40,260 (19.8)	162,890 (80.2)
95% LCL – UCL	36,930 – 41,590	152,454 – 167,526	37,693 – 42,416	158,483 – 174,066	40,857 – 45,803	160,084 – 175,996	42,014 – 47,206	168,354 – 185,106	37,941 – 42,579	155,029 – 170,751
Black	8,275 (23.0)	27,765 (77.0)	8,560 (22.9)	28,850 (77.1)	9,520 (23.9)	30,335 (76.1)	9,630 (23.7)	31,070 (76.3)	9,140 (23.7)	29,370 (76.3)
95% LCL – UCL	7,607 – 8,943	25,889 – 29,641	7,838 – 9,282	26,979 – 30,721	8,729 – 10,311	28,262 – 32,408	8,849 – 10,411	28,891 – 33,249	8,423 – 9,857	27,334 – 31,406
Hispanic	4,650 (20.6)	17,915 (79.4)	4,630 (19.5)	19,170 (80.5)	5,195 (19.9)	20,915 (80.1)	5,135 (19.7)	20,930 (80.3)	4,960 (20.5)	19,250 (79.5)
95% LCL – UCL	4,158 – 5,142	16,207 – 19,623	4,131 – 5,129	17,393 – 20,947	4,576 – 5,814	18,837 – 22,993	4,599 – 5,671	18,911 – 22,949	4,400 – 5,520	17,468 – 21,032
Asian/Pacific Islander	1,505 (16.5)	7,590 (83.5)	1,575 (16.0)	8,250 (84.0)	1,625 (16.2)	8,400 (83.8)	1,695 (15.4)	9,305 (84.6)	1,690 (16.0)	8,870 (84.0)
95% LCL – UCL	1,265 – 1745	6,730 – 8,450	1,294 – 1,856	7,207 – 9,293	1,365 – 1,885	7,340 – 9,460	1,444 – 1,946	8,147 – 10,463	1,435 – 1,945	7,878 – 9,862
Native American	215 (18.0)	980 (82.0)	280 (23.4)	915 (76.6)	240 (19.0)	1,020 (81.0)	240 (19.8)	970 (80.2)	280 (22.9)	945 (77.1)
95% LCL – UCL	139 – 291	737 – 1,223	192 – 368	721 – 1,109	160 – 320	806 – 1,234	150 – 330	778 – 1162	197 – 363	752 – 1138
Other	2,115 (23.1)	7,055 (76.9)	2,275 (22.2)	7,970 (77.8)	1,900 (19.2)	8,005 (80.8)	2,035 (22.0)	7,225 (78.0)	1,770 (21.0)	6,665 (79.0)
95% LCL – UCL	1,471 – 2759	5,144 – 8,966	1,335 – 3,215	4,692 – 11,248	1,204 – 2,596	4,911 – 11,099	1,695 – 2,375	6,082 – 8,368	1,493 – 2,047	5,831 – 7,499
Insurance payer, n										
Medicare	26,740	115,350	28,200	123,745	30,215	125,910	30,645	132,215	28,575	121,005
95% LCL – UCL	25,403 – 28,077	111,107 – 119,593	26,778 – 29,622	119,027 – 128,463	28,745 – 31,685	121,246 – 130,574	29,103 – 32,187	127,091 – 137,339	27,153 – 29,997	116,402 – 125,608
Medicaid	8,395	27,335	8,100	28,450	8,465	29,180	9,070	28,875	8,240	27,875
95% LCL – UCL	7,802 – 8,988	25,785 – 28,885	7,524 – 8,676	26,823 – 30,076	7,840 – 9,090	27,525 – 30,835	8,441 – 9,699	27,274 – 30,476	7,644 – 8,836	26,279 – 29,471
Private insurance	19,920	76,090	19,820	74,755	20,930	74,540	21,345	76,715	19,240	71,170
95% LCL – UCL	18,292 – 21,548	71,075 – 81,105	18,199 – 21,441	69,601 – 79,909	19,353 – 22,507	69,467 – 79,613	19,631 – 23,059	71,489 – 81,941	17,760 – 20,720	66,361 – 75,979
Self-pay	1,325	5,195	1,375	5,415	1,620	5,665	1,735	6,335	1,690	5,640
95% LCL – UCL	1,114 – 1,536	4,556 – 5,834	1,160 – 1,590	4,753 – 6,077	1,398 – 1,842	5,033 – 6,297	1,485 – 1,985	5,581 – 7,089	1,460 – 1,920	4,960 – 6,320
No charge	155	495	120	410	95	415	115	435	90	490
95% LCL – UCL	88 – 222	320 – 670	44 – 196	239 – 581	45 – 145	286 – 544	67 – 163	299 – 571	44 – 136	337 – 643
Other	1,690	6,665	1,710	6,470	1,730	6,650	1,705	6,865	1,615	6,660
95% LCL – UCL	1,420 – 1,960	5,935 – 7,395	1,486 – 1,934	5,838 – 7,102	1,500 – 1,960	5,986 – 7,314	1,695 – 2,375	6,196 – 7,534	1,383 – 1,847	6,042 – 7,278

LCL, lower confidence limit; UCL, upper confidence limit.
